# Rapid Antigen Test for Pandemic (H1N1) 2009 Virus

**DOI:** 10.3201/eid1605.091574

**Published:** 2010-05

**Authors:** Bram M.W. Diederen, Dick Veenendaal, Ruud Jansen, Bjorn L. Herpers, Eric E.J. Ligtvoet, Ed P.F. IJzerman

**Affiliations:** Regional Laboratory of Public Health Haarlem, Haarlem, the Netherlands

**Keywords:** Influenza, pandemic (H1N1) 2009, rapid antigen testing, point of care test, RT-PCR, viruses, expedited, letter

**To the Editor:** Drexler et al. recently compared the sensitivity of the BinaxNOW Influenza A & B Rapid Test (BinaxNOW; Inverness Medical, Cologne, Germany) with that of a real-time reverse transcription–PCR (RT-PCR) assay specific for influenza A pandemic (H1N1) 2009 virus (*1*). Of 1,838 clinical specimens tested, 221 were confirmed as positive for pandemic (H1N1) 2009 by RT-PCR. When 144 of these 221 specimens were evaluated by using the BinaxNOW, results were positive for only 16 (11%).

At onset of the pandemic, we evaluated the first 135 nasopharyngeal aspirates submitted to the Regional Laboratory of Public Health Haarlem, the Netherlands. We compared the performance of the BinaxNOW for diagnosing influenza A (H1N1) virus by using molecular detection of influenza virus as the reference standard. Samples were analyzed with a general influenza A assay targeting the matrix gene (the RespiFinder assay) (PathoFinder B.V., Maastricht, the Netherlands [*2*]) and a pandemic (H1N1) 2009–specific RT-PCR assay targeting the neuraminidase gene (*3*). We tested 135 patient samples (76 from male patients); mean age of patients was 32 years (range 0–81 years). Samples from 38 (28%) patients had positive results in both RT-PCRs, and samples from 97 (72%) patients had negative results in the matrix gene RT-PCR and neuraminidase RT-PCR assays. Sensitivity and specificity were estimated to be 47% (18/38, 95% confidence interval [CI] 32%–62%) and 95% (92/97, 95% CI 88%–98%), respectively, for the BinaxNOW antigen test. Patients’ ages did not significantly differ between rapid test–positive and –negative results.

Our results largely agree with those of Vasoo et al. (*4*) and the Centers for Disease Control and Prevention (*5*). Those studies determined that the sensitivity of the BinaxNOW compared with nucleic acid amplification tests is ≈40%. The lower sensitivity observed by Drexler et al. (*1*) might be because of differences in study type (retrospective evaluation compared with a prospective cohort in our study), sample size, technical factors (with regard to specimen collection, specimen transport, and specimen storage), differences in the test kit, and differences between individual patients (multiple categories of age and stages of illness, differences in virus shedding).

Many clinicians are not aware of the performance of specific test devices and rely on test results to make clinical decisions. Because negative results cannot rule out influenza, this test is of little use in a clinical setting without appreciation of the limitations of the test. However, because the BinaxNOW has reasonable specificity, it might prove useful in clinical or epidemiologic situations in which test sensitivity is not critical, e.g., in facility outbreaks in which multiple specimens are collected to rapidly identify the causative organism.
