# Supplementary material for: Rapid Antigen Test for Pandemic (H1N1) 2009 Virus
Source: Emerg Infect Dis. 2010 May;16(5):897–8. doi: 10.3201/eid1605.091574 (PMC2954002; doi:10.3201/eid1605.091574)
Supplement: Related texts

# Letter

**DOI:** 10.3201/eid1605.100326

**Published:** 2010-05

**Authors:** Jan Felix Drexler, Christian Drosten, Anna Maria Eis-Hübinger

**Affiliations:** University of Bonn Medical Centre, Bonn, Germany

## In Response

We read with interest the report by Diederen et al. (*1*) showing a 47% sensitivity of the BinaxNOW (Inverness Medical, Cologne, Germany) antigen-based rapid influenza diagnostic test (RIDT) for the clinical detection of pandemic (H1N1) 2009 virus. We agree that RIDTs may be of little benefit in situations where a timely diagnosis by reverse transcription–PCR (RT-PCR) or optimized direct fluorescent antibody tests can be achieved.

Our recent study yielded even lower sensitivity for RIDT: 11.1% (*2*). RIDT sensitivity is greatly influenced by differences in the level of virus shedding between children and adults, making studies difficult to compare (*3*). In general, age profiles and virus concentrations should be provided and considered when comparing cohorts examined by any virus detection method. Moreover, quality and origin of specimens can influence the sensitivity of RT-PCR– and antigen-based tests. One important example is the use of flocked swabs for collecting respiratory samples. Under optimal conditions, for instance, a direct fluorescent antibody test was recently shown to yield high diagnostic sensitivity comparable with that of RT-PCR for pandemic (H1N1) 2009 virus (*4*). Another critical factor, especially for RIDT, may be the compatibility of test monoclonal antibodies with the novel virus. Lower sensitivities of such tests for pandemic (H1N1) 2009 virus in comparison with seasonal influenza viruses have been reported (*3**,**5*). Adaptation of RIDT antibody selection to pandemic (H1N1) 2009 virus may thus be necessary. Finally, we would like to emphasize the medical risks associated with use of RIDTs by untrained operators, e.g., lesions from inadequate sampling and false interpretation of test results. Such use may be specifically promoted by ready availability of such tests on the Internet or at pharmacies.

## References

[1] Diederen BMW, Veenendaal D, Jansen R, Herpers BL, Ligtvoet EEJ, IJzerman EPF. Rapid antigen test for pandemic (H1N1) 2009 virus [letter]. Emerg Infect Dis. 2010;16:897–8.2040940410.3201/eid1605.091574PMC2954002

[2] Drexler JF, Helmer A, Kirberg H, Reber U, Panning M, Muller M, Poor clinical sensitivity of rapid antigen test for influenza A pandemic (H1N1) 2009 virus. Emerg Infect Dis. 2009;15:1662–4.1986106910.3201/eid1510.091186PMC2866420

[3] Centers for Disease Control and Prevention. Evaluation of rapid influenza diagnostic tests for detection of novel influenza A (H1N1) virus—United States, 2009. MMWR Morb Mortal Wkly Rep. 2009;58:826–9.19661856

[4] Pollock NR, Duong S, Cheng A, Han LL, Smole S, Kirby JE. Ruling out novel H1N1 influenza virus infection with direct fluorescent antigen testing. Clin Infect Dis. 2009;49:e66–8. 10.1086/64450219681710PMC2768282

[5] Kok J, Blyth CC, Foo H, Patterson J, Taylor J, McPhie K, Comparison of a rapid antigen test with nucleic acid testing during cocirculation of pandemic influenza A/H1N1 2009 and seasonal influenza A/H3N2. J Clin Microbiol. 2010;48:290–1. 10.1128/JCM.01465-0919889892PMC2812250
